# Peptidylarginine deiminase IV promotes the development of chemoresistance through inducing autophagy in hepatocellular carcinoma

**DOI:** 10.1186/2045-3701-4-49

**Published:** 2014-08-26

**Authors:** Tingting Fan, Changsong Zhang, Ming Zong, Qiudong Zhao, Xue Yang, Chong Hao, Hui Zhang, Shanshan Yu, Jinhu Guo, Ruhan Gong, Shasha Fan, Lixin Wei, Lieying Fan

**Affiliations:** Department of Clinical Laboratory, Shanghai East Hospital, Tongji University Medical School, No. 150, Jimo Road, Shanghai, 200120 China; Clinical Oncology Laboratory, Changzhou cancer Hospital of Soochow University, Changzhou, 213001 China; Tumor Immunology and Gene Therapy Center, Eastern Hepatobiliary Surgery Hospital, Second Military Medical University, 225 Changhai Road, Shanghai, 200438 China

**Keywords:** PADI4, Autophagy, Hepatocellular Carcinoma, Chemoresistance

## Abstract

**Background:**

Peptidylarginine deiminase IV (PADI4) is widely distributed in several tissues and the expression is correlated with many pathological processes. Chemotherapy remains a major treatment alternatively to surgery for a large number of patients at the advanced stage of hepatocellular carcinoma (HCC). However, the role of PADI4 in the chemoresistance of HCC has not been identified.

**Methods:**

MTT and PI/Annexin V assay were employed to examine the proliferation and apoptosis of HCC cell lines. The expression of MDR1 is detected by Realtime PCR. GFP tagged LC3 expression vector and electron microscopy are utilized to demonstrate the occurrence of autophagy.

**Results:**

We observed that the elevated PADI4 expression is associated with chemoresistance in HCC patients with TACE after surgery. In addition, we found that overexpression of PADI4 in HCC cell lines lead to the resistance to chemotherapeutic agents in vitro and in vivo. Interestingly, the HCC cells that overexpressed PADI4 were observed to undergo autophagy which was known as a protective mechanism for cells to resist the cell tosicity from chemotherapy. Autophagy inhibitor could effectively restore the sensitivity of HCC cells to chemotherapy in vitro and in vivo.

**Conclusions:**

These results indicate that PADI4 may induce chemoresistance in HCC cells by leading autophagy.

**Electronic supplementary material:**

The online version of this article (doi:10.1186/2045-3701-4-49) contains supplementary material, which is available to authorized users.

## Background

Peptidylarginine deiminases (PADs) enzymes distributed widely in mammals and their function is closely associated with the development and differentiation of the cells [1]. PADs can convert protein Arg residues to citrulline in a calcium and sulfhydryl group dependent manner, which play an important role in several physiological and pathological processes such as gene expression regulation, epithelial terminal differentiation and apoptosis [2].

There are five types of PAD enzymes in human and mouse, including PADI1-4 and PAD6 [1, 3]. PADI4 has been reported to express in several type of cells, including embryonic stem cells, leukocytes and breast cancer cells. Furthermore, PADI4 is also involved in the etiology of cancers and rheumatoid arthritis in human [4–6]. The results from immunohistochemistry demonstrates that PADI4 is expressed not only in various adenocarcinoma, but also in some non-adenocarcinoma tumors such as bone osteosarcoma, liver cholangiocellular carcinoma, liver hepatocellular carcinoma and lung squamous carcinoma [7]. These results indicates that PADI4 may play an important role in tumorigenesis [8].

Hepatocellular carcinoma (HCC) is a common malignant tumor in China, which surgical resection is considered as the most effective treatment. Chemotherapy remains to be a major treatment alternatively to surgery for a large number of patients at the advanced stage of HCC. However, chemoresistance in HCC is still a prominent obstacle for effective treatment of HCC with chemotherapy. Therefore, the underlying molecular mechanisms of HCC chemoresistance and how to overcome them is still a critical issue for us to identify. The role of PADI4 for chemoresistance of HCC cells has not been identified. In this study, we will investigate the role of PADI4 for chemoresistance in HCC and the potential mechanism will could be explored.

## Methods

### Patients and tissue specimens

Specimens of HCC tissues were obtained from 120 HCC patients who underwent hepatic resection at Eastern Hepatobiliary Surgery Hospital of Second Military Medical University from February 1998 to December 2010. These patients included 90 males and 30 females with a median age of 51 years (range: 31–73). All tumors were histologically diagnosed as HCC according to the Edmondson-Steiner classification system. In all cases, H&E-stained slides were re-examined independently by three experienced pathologists without any knowledge of clinical data. Written informed consent was obtained from each patient, and the protocol of the study was approved by the ethics committee of Second Military Medical University.

### Cell lines and reagents

HCC cell lines including SMMC-7721 cells and Hep-G2 cells were cultured in Dulbecco’s modified Eagle’s medium (DMEM) (GIBCO, Invitrogen) with 10% fetal bovine serum (FBS) at 37°C in a humidified atmosphere containing 5% CO_2_. The chemotherapeutic agents: cisplatin and 5-fluorouracil (5-FU) were purchased from Qilu Pharmaceutical Co., Ltd. (JiNan, Shandong, China). 3-methyladenine (3-MA) (Sigma-Aldrich, Cat.M9281) was used at 10 mM.

### Adenoviral vectors

The adenoviral vector expressing PADI4 under a cytomegalovirus promoter promoter—AdPADI4 was used to overexpress PADI4 in HCC cell lines. Ad-Vector (control adenovirus) infected group was used as controls in the subsequent experiments.

### HCC cell treatment

To investigate the effect of PADI4 on the chemotherapy sensitivity of HCC cells, the cells were infected with AdPADI4 and then collected for the determinations of cell viability, apoptosis and the autophagy associated genes expression.

### Quantitative real-time PCR analysis for mRNA expression

RNA was isolated from tumor tissues, and blood samples. In brief, total RNA was extracted with TRIzol reagent, according to the protocol provided by the manufacturer. The quantity and quality of the RNA samples were measured carefully by spectrophotometer and electrophoresis. The first-strand cDNA was synthesised from 2 μg of total RNA. Primer sequences of PADI4 for reverse transcription-PCR (RT-PCR) reaction were forward (5′-CACAGCTCTGGTTGGCTTCA-3′) and reverse (5′-CTGCACGTCCTTCAGCATCA-3′) [9]. Primer sequences of MDR1 for reve rse transcription-PCR (RT-PCR) reaction were forward (5′- CTGGTTTGATGTGCACGATGTTGG-3′) and reverse (5′- TGCCAAG-ACCTCTTCAGCTACTG-3′) [10]. Quantitative real-time PCR (qPCR) were carried out by using the Mx3000P QPCR System (Stratagene, USA). As an internal control for qPCR, β-actin mRNA expression was amplified from the same cDNA samples. All results were normalized to β-actin amplification. *C*_T_ values for triplicate reactions were averaged and relative expression was determined with the comparative *C*_T_ method, using average *C*_T_ values.

### MTT colorimetric assay

In order to examine the effect of PADI4 on the chemosensitivity of HCC cells, HCC cells were seeded in 96-well plates at a density of 1 × 10^4^ cells/well and cultured in the medium containing chemotherapeutic agents with Ad-PADI4. The cell viability in each well was examined by a MTT (5 mg/ml, Sigma, Cat.M2003) colorimetric assay. The optical density (OD) value at 490 nm of each sample was measured using a plate reader. The data was expressed as mean ± SD.

### Cell apoptosis assay

HCC cells (2 × 10^5^ cells/well) were cultured in 6-well plates to 70–80% confluence. The cells were then treated with chemotherapeutic agents for 8 h in the absence and presence of MSC conditioned medium. In a subset of experiments, 3-MA (5 mM) was used to block autophagy. PI/Annexin V-PE assay was used to measure apoptotic cells by flow cytometry according to the manufacturer’s instruction (Keygen Biotech. Co., Ltd, Nanjing, Jiangsu, China, Cat.KGA108). Briefly, cells collected by trypsinization were washed trice with ice cold phosphate-buffered saline (PBS). Cells were then incubated in 300 μL of 1× binding buffer containing 5 μL Annexin V and 5 μL PI for 30 min at room temperature in the dark. Apoptosis of cells was measured on a BD FACSAria flow cytometer (Becton Dickinson, Lincoln Park, NJ). At least 30,000 gated events were acquired from each sample. Results are expressed as the percentage of apoptotic cells (PI and Annexin V positive) in the gated cell population.

### Transient transfection and identification of autophagy

GFP tagged LC3 expression vector has recently been utilized to demonstrate the occurrence of autophagy. SMMC-7721 and Hep-G2 cells were seeded (1 × 10^4^ cells/well) in 96-well plates overnight. GFP-LC3 expressing plasmids were transiently transfected into cells using the Fugene HD transfection reagent (Roche, Cat.04709705001) according to the manufacturer’s instruction. After being cultured for 24 h to ensure expression of GFP-LC3, the cells were treated with Ad-PADI4 for 8 h. At the end of each experiment, autophagy was detected by counting the percentage of cells with GFP-LC3-positive dots under fluorescence microscope (Olympus IX71). A minimum of 200 cells were counted in each sample. The experiment was conducted in triplicate.

### Electron microscopy

HCC cells were sequentially fixed with 2.5% glutaraldehyde acid in 0.1 M PBS buffer (pH 7.4) for 2 h, incubated in 1% osmium tetroxide in 0.1 M PBS buffer (pH 7.4) for 2–3 h, dehydrated in solutions of ethanol and acetone, embedded in Araldite, and finally solidified. Sections (50–60 nm) were cut on a LKB-I ultramicrotome and picked up on copper grids. After being post-stained with uranyl acetate and lead citrate, sample sections were observed with a Philips CM-120 TEM (Philips).

### Western blot analysis

HCC cell lines were washed in PBS and lysed in RIPA buffer with 1 mM PMSF on ice. Cell lysates were centrifuged (12,000 rpm, 10 min) at 4°C, the protein supernatant was transferred into new tubes. The concentration of the protein samples was determined with BCA Protein Assay Kit (Pierce, USA). A 20 μg sample of the total protein was resolved using 12% SDS-PAGE and transferred onto PVDF membranes. The membranes were blocked in Tris-buffered saline containing Tween 20 (TBST) with 5% nonfat milk at room temperature for 2 h. Primary antibodies to detect PADI4(1:1000, Abcam, USA) and LC-3(1:1000, Novus Biologicals, Inc) were incubated overnight with the membranes at 4°C. Membranes were incubated with horseradish peroxidase (HRP)-conjugated anti-rabbit secondary antibodies (1:8000, Dako, USA), and proteins were detected by enhanced chemiluminescence (ECL) (Beyotime, USA). GAPDH was used as the internal control to normalize the loading materials.

### Animal model

All procedures involving animals were performed in accordance with the institutional animal welfare guidelines of Second Military Medical University. Subcutaneous implantation of HCC cells was performed in armpit areas of nude mice. Mice were examined three times per week. Tumor growth was evaluated by measuring the length and width of the tumor mass. Animals were sacrificed and tumors were removed at the end of the experiment. Tumor masses were weighed and analyzed by histology.

### Short Hairpin RNA (shRNA) synthesis and transient transfection

The shRNA sequences of PADI4, Atg7 and Beclin7 were designed using Oligoengine software and verified by nucleotide BLAST searches. The candidate sequences and a scrambled sequence with no significant homology were listed in Additional file 1: Table S1. The recombinant virus was packaged using Lentivector Expression Systems (Shanghai GeneChem, Shanghai). Cells (1–3 × 10^6^) growing to 50%-60% confluence in 10 cm petri dishes were transfected with shRNA sequences. Cells were observed under a fluorescence microscope and harvested 48 h after transfection.

### Statistical analysis

All data were generated without knowledge of the clinical status of the samples analyzed by SPSS 18.0 software (SPSS, Inc., Chicago, USA). Comparison was done with t test (unpaired or paired). All P values presented were two-sided, and a P value of less than 0.05 was considered statistically significant.

## Results

### The elevated PADI4 expression is associated with chemoresistance in HCC with Transcatheter arterial chemoembolization (TACE)

**Figure 1:**
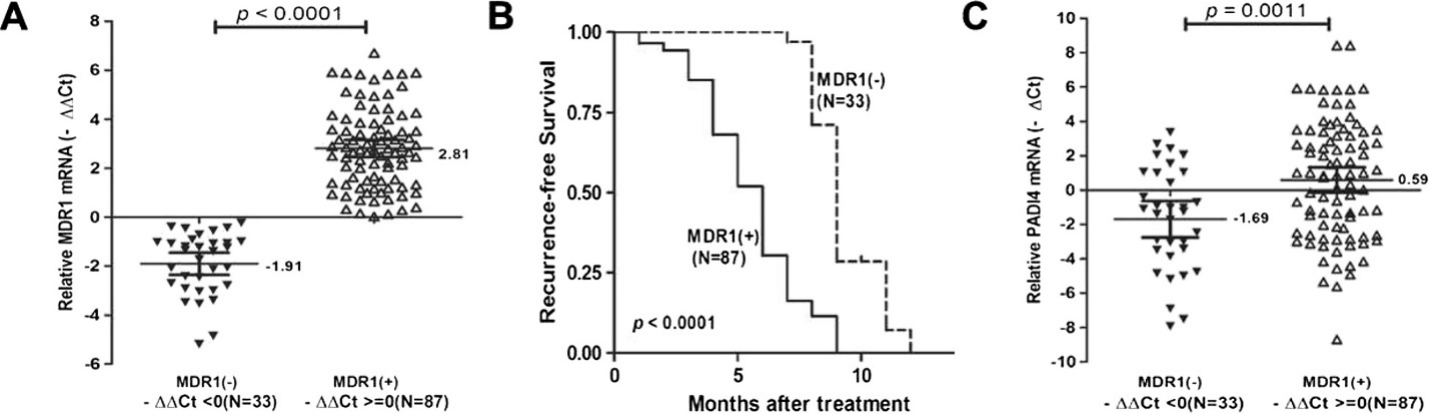
**The elevated PADI4 mRNA expression in MDR1(+) HCC patients with TACE. (A)** The MDR1 mRNA expression were detected in blood samples before and after TACE. - ΔΔCt, -(ΔCt_before TACE_ – ΔCt_after TACE_); MDR(+):- ΔΔCt > =0; MDR(-):- ΔΔCt < 0. **(B)** The progression-free survival (PFS) after transcatheter arterial chemoembolization (TACE) in partial hepatectomy for hepatocellular carcinoma determined by Kaplan-Meier analysis. **(C)** The PADI4 mRNA expression in HCC tumor tissues associated with chemoresistance. - ΔΔCt, -(ΔCt_before TACE_ – ΔCt_after TACE_); MDR(+):- ΔΔCt > =0; MDR(-):- ΔΔCt < 0.

We collected 5 ml vein blood samples before TACE and after TACE in HCC patients with partial hepatectomy. Chemoresistance were detected by MDR1 mRNA expression in blood samples by using Realtime-PCR. We found that elevated expression of MDR1 after TACE was detected in 72.5% of HCC patients (87/120), and lower expression of MDR1 was detected in 27.5% of HCC patients (33/120) after TACE. Here, MDR1 (+) group means elevated expression of MDR1, and MDR1 (-) group means lower expression of MDR1 after TACE treatment for HCC patients with partial hepatectomy. According to the MDR1 results, all 120 HCC patients were divided into two groups: the chemoresistance group--MDR1 (+) group, and chemosensitivity group--MDR1 (-) group (Figure 1A). However, there are no significant difference in clinicopathologic variables between MDR1 (+) group and MDR1 (-) group (Additional file 1: Table S2).

Time zero of recurrence-free survival of patients treated with TACE is the date of TACE. All patients underwent ultrasound, computed tomography scan or magnetic resonance imaging of the abdomen once every 2 months after treatment. Kaplan–Meier analysis indicated that the recurrence-free survival of MDR1 (+) group was shorter than MDR1 (-) group (median: 6 months vs 9 months, respectively; *P* < 0 · 0001; HR: 6.339, 95CI%: 3.742-10.74, Figure 1B). Interestingly, we found that PADI4 mRNA expression of tumor tissues was higher in MDR1 (+) group (Mean _-∆Ct_ ± SD = 0.59 ± 0.37) than that in MDR1 (-) group (Mean _-∆Ct_ ± SD = -1.69 ± 0.53). PADI4 expression of HCC tissues was significantly difference between chemoresistance patients and chemosensitivity patients (*p* = 0.0011; Figure 1C).

### PADI4 enhanced chemoresistance of hepatocellular carcinoma cells

**Figure 2:**
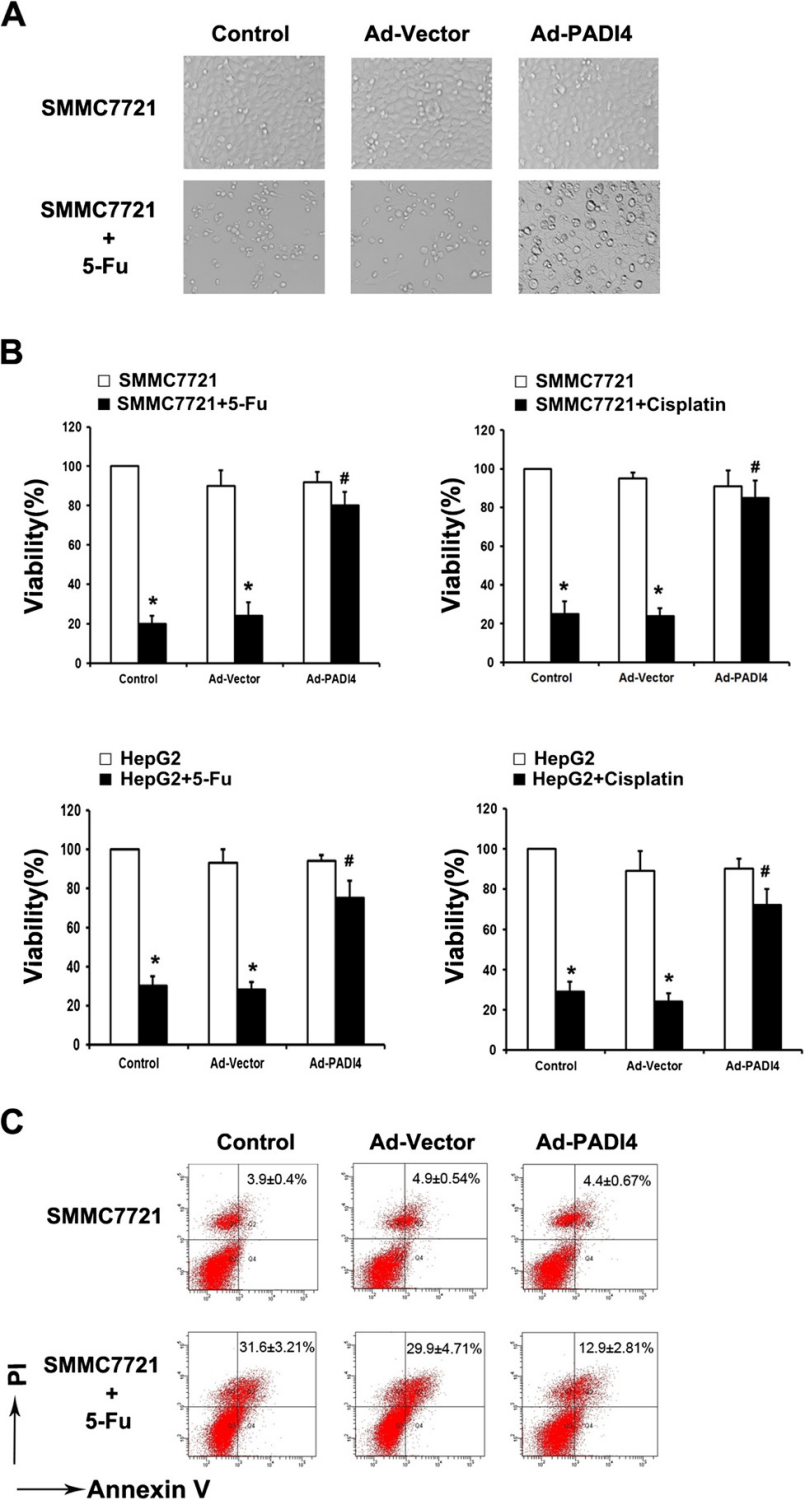
**PADI4 enhanced chemoresistance of hepatocellular carcinoma cells** ***in vitro.*** **(A)** SMMC-7721 cells were treated with 5-Fu (120 μg/mL) with overexpression of PADI4 or not. The morphology of the cells was observed by microscope. **(B)** PADI4 was overexpressed in SMMC-7721 or HepG2 cells and the cells (1 × 10^4^/well) were cultured in a 96-well plate with an existence of 5-Fu (120 μg/mL) or cisplatin (8 μg/mL) for 24 hours. MTT was employed to examine the viability of SMMC-7721 or Hep-G2 cells. **(C)** PADI4 was overexpressed in SMMC-7721 cells and the cells were cultured in a 6-well plate with an existence of 5-Fu (120 μg/mL) for 24 hours. Flow cytometry was used to measure apoptosis of the cells. (*Compared with the group that untreated with chemotherapy drugs, P < 0.05; #Compared with the group that treated with chemotherapy drugs, P < 0.05)

**Figure 3:**
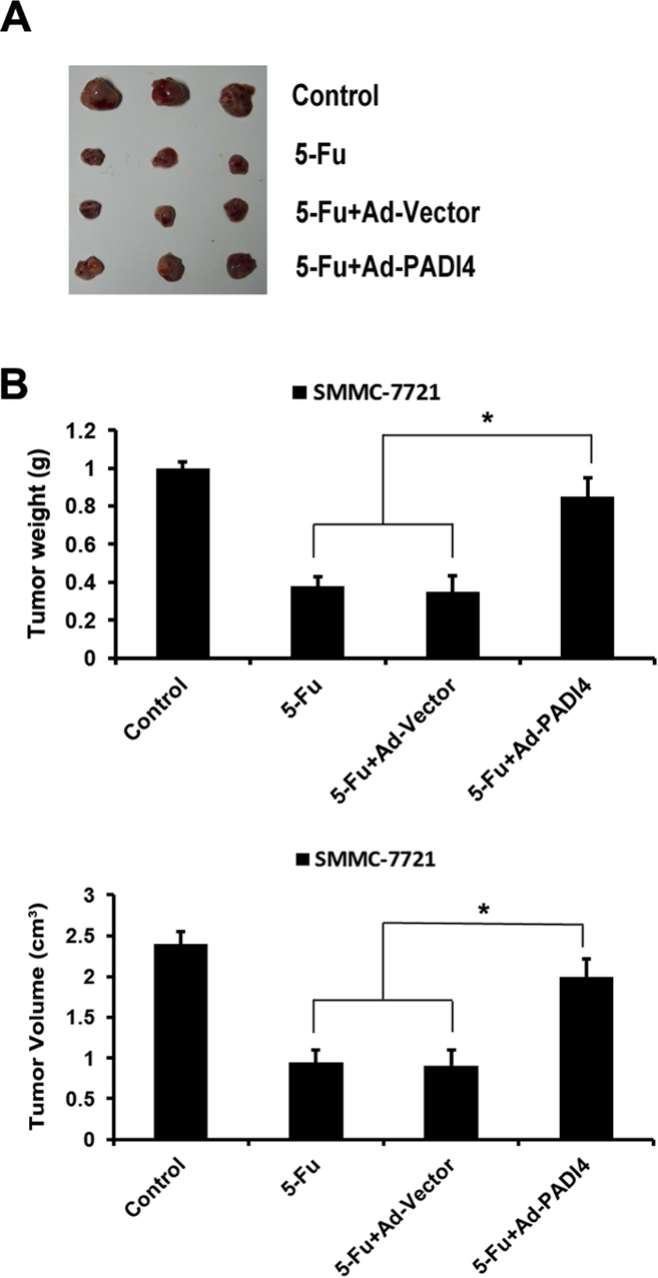
**PADI4 enhanced chemoresistance of hepatocellular carcinoma cells** ***in vivo.*** **(A)** PADI4 was overexpressed in SMMC-7721 cells and then the cells (5 × 10^6^) to perform subcutaneous administration in the nude mice armpit area. After implantation, recipients were injected in tumor *in situ* with 5-Fu (30 mg/kg) every 3 days. After 27 days of implantation, the animals were sacrificed and tumors were dissected. **(B)** The tumor weight and volume of each groups were measured after been removed from the mice. (*P < 0.05)

In order to investigate the influence of PADI4 on the chemoresistance in HCC cells, we observed the effect of chemotherapeutic agent 5-Fu on morphological changes of HCC cell line-SMMC7721 cells. The expression of PADI4 in HCC cell lines was examined by western blot and Ad-PADI4 was used to overexpress PADI4 in HCC cell lines (Additional file 2: Figure S1). As shown in Figure 2A, 5-Fu could effectively inhibit the growth of SMMC7721 cells. Once PADI4 was overexpressed in SMMC7721 cells, the inhibition effect induced by 5-Fu was attenuated. We also observed the role of PADI4 on the proliferation of SMMC7721 and Hep-G2 cells when they were exposed to chemotherapeutic agent. The results demonstrated that overexpression of PADI4 significantly improved the chemoresistance capability in hepatocarcinoma cell lines compared with control groups (Figure 2B). In addition, we detected the effect of PADI4 on the apoptosis of HCCs when they were exposed to chemotherapeutic agent. The results showed that PADI4 effectively decreased the apoptosis of SMMC7721 cells induced by 5-Fu (Figure 2C). We further examined the PADI4-induced chemoresistance in HCC cells in the nude mouse model. As shown in Figure 3A and B, compared with control groups, PADI4 induced the resistance to chemotherapy in HCC cells *in vivo*.

Taken together, these data suggested that overexpression of PADI4 could effectively induce the chemoresistance in HCC cells.

### PADI4 induced autophagy in hepatocellular carcinoma cells

**Figure 4:**
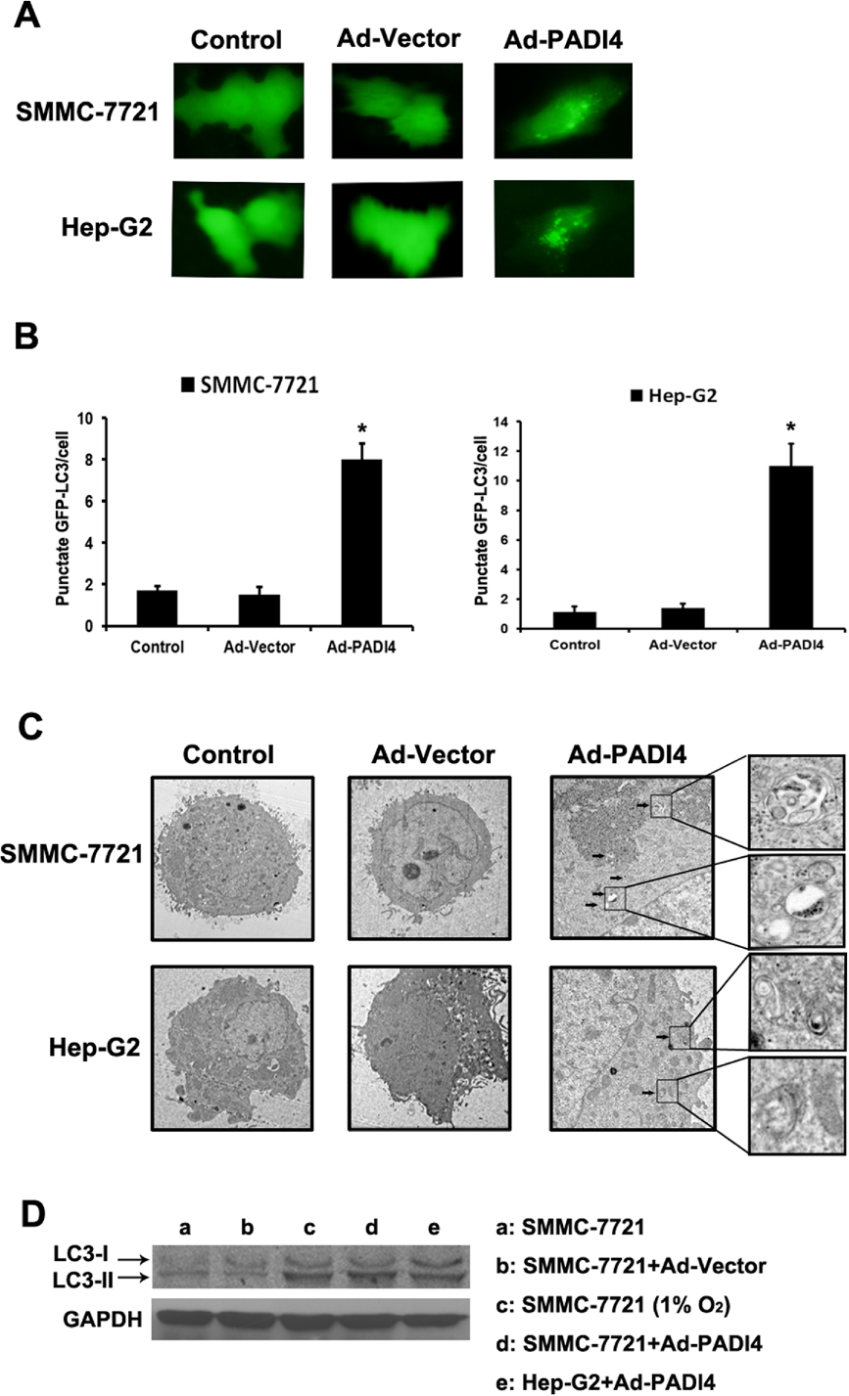
**PADI4 induced autophagy in hepatocellular carcinoma cells. (A)** GFP-tagged LC3 plasmid was transfected into SMMC-7721 cells, after 24 hours transfection, the cells were incubated with Ad-PADI4. Fluorescence microscope was used to observe the punctate GFP-LC3 in the cytoplasm. **(B)** The number of punctate GFP-LC3 in each cell of SMMC-7721 and HepG2 was counted and at least 100 cells were included for each group. **(C)** Electron micrographs was exployed to examine the autophagic vacuoles ultrastructure in the cytoplasm of SMMC-7721 and HepG2 cells which PADI4 were overexpressed. Magnification, ×10,000. **(D)** Western blot was used to analysis the expression of LC3-I and LC3-II in SMMC7721 and HepG2 cells. a-d: SMMC-7721 in different treatment conditions; e: HepG2 treated with Ad-PADI4. GAPDH expression was used as control.

Autophagy has been reported to contribute to chemoresistance in HCC cells [11]. Therefore, we speculated that PADI4 might lead to an incidence autophagy in HCC cells. A vector that expresses encoding GFP-LC3 was transmitted into SMMC7721 cells to detect the occurrence of autophagy. We determined the incidence of autophagy in HCC cells when the punctuate GFP fluorescence was observed in a diffused GFP fluorescence. The results showed that SMMC7721 with overexposed PADI4 exhibited a high percentage of punctuate GFP, which indicated the occurrence of autophagy, while the fluorescence in control groups remained to be diffused (Figure 4A and B). Transmission electron microscopy was employed to confirm the autophagy in SMMC7721 cells. As shown in Figure 4C, SMMC7721 cells with overexpressed PADI4 demonstrated a marked autophagosomes accumulation, which indicated the incidence of autophagy. Furthermore, we examined the expression of LC3-I and LC3-II in HCC cells by western blot. The level of LC3-I and LC3-II increased in PADI4 overexpression groups and in hypoxia treated group, which indicated that PADI4 could induce autophagy in HCC cells (Figure 4D).

### Inhibition of autophagy restored the sensitivity of HCC cells to chemotherapy

**Figure 5:**
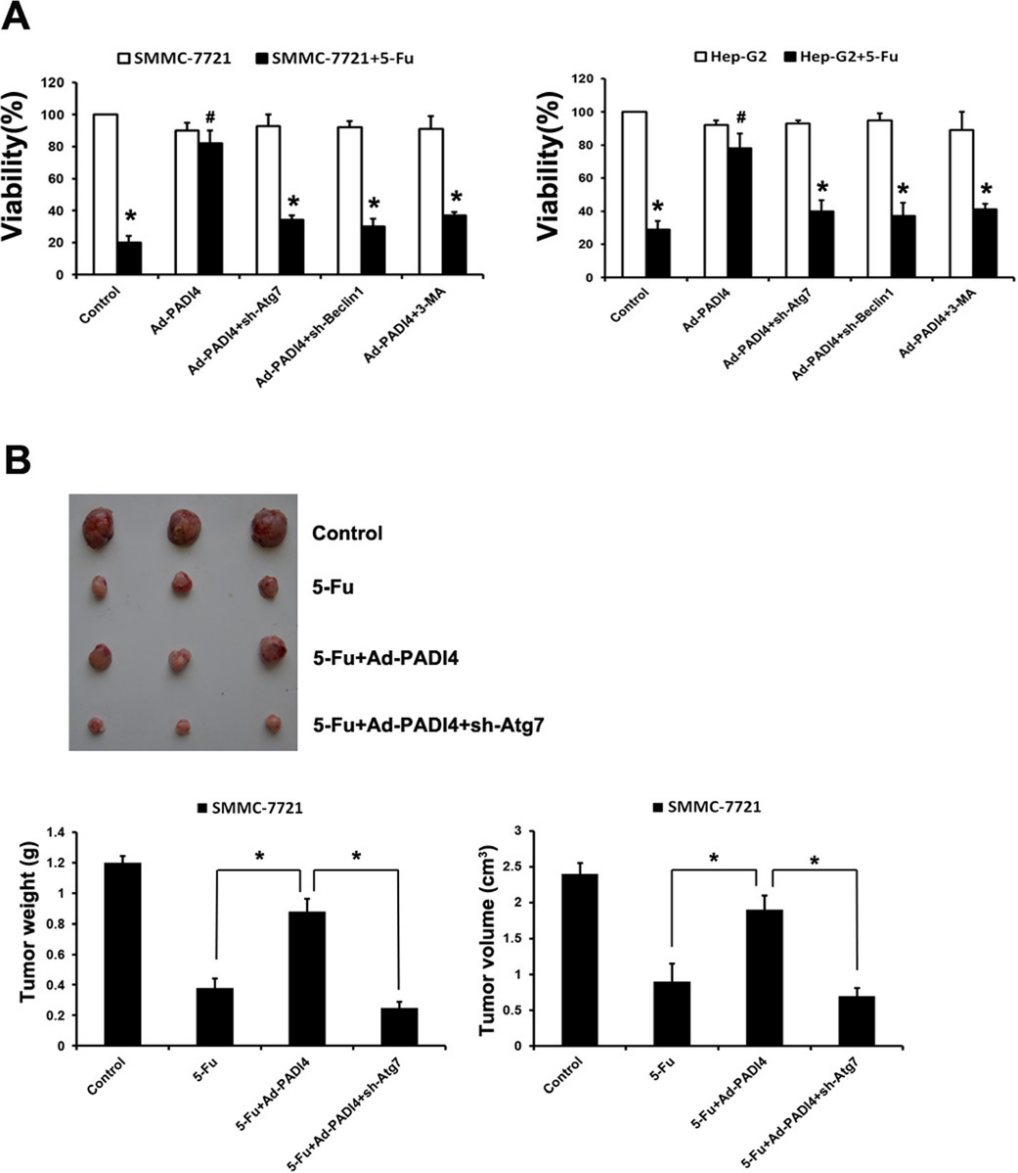
**Inhibition of autophagy restored the sensitivity of HCC cells to chemotherapy. (A)** SMMC-7721 and Hep-G2 cells (1 × 10^4^/well) that overexpressed PADI4 were cultured in a 96-well plate with an existence of 5-Fu (120 μg/mL) for 24 hours. The occurrence of autophagy was inhibited by autophagy inhibitor-3-MA or sh-Atg7 and sh-Beclin1. MTT was used to detect the viability of the cells. **(B)** PADI4 was overexpressed in SMMC-7721 cells. The occurrence of autophagy was inhibited by sh-Atg7. Then the cells (5 × 10^6^) were performed subcutaneous administration in the nude mice armpit area. After implantation, recipients were injected in tumor *in situ* with 5-Fu (30 mg/kg) every 3 days. After 27 days of implantation, the animals were sacrificed and tumors were dissected. The tumor weight and volume of each groups were measured after been removed from the mice. (*Compared with the group that untreated with chemotherapy drugs, P < 0.05; #Compared with the group that treated with chemotherapy drugs, P < 0.05)

In order to confirm the role of autophagy in enhancing the chemoresistance of HCC cells, we observed the recovery of HCC cell sensitivity to chemotherapy leading by autophagy inhibitors. The 3-MA is used as autophagy inhibitor, which blocks the formation of autophagosomes [12]. As shown in Figure 5, the results demonstrated that 3-MA could significantly restore the chemotherapeutic sensitivity in HCC cells. Beside that, another autophagy inhibitor-CQ demonstrated a same effect as 3-MA (Additional file 3: Figure S2). Furthermore, we also employed shRNA-ATG7 or shRNA-Beclin1 to inhibit autophagy in HCC cells. As shown in Additional file 4: Figure S3, shRNA-ATG7 and shRNA-Beclin1 could effectively inhibit the upregulation of ATG7 and Beclin1 in HCC cells during cultured in hypoxia condition. The inhibition of ATG7 or Beclin1 respectively in HCC cells effectively diminished the protection of autophagy on chemoresistance*.*

## Discussion

Hepatocellular carcinoma (HCC) is a common malignant tumor in China, which surgical resection is considered as the most effective treatment. Chemotherapy remains to be a major treatment alternatively to surgery for a large number of patients at the advanced stage of HCC. However, chemoresistance in HCC cells is still a prominent obstacle for effective treatment of HCC with chemotherapy.

Multiple drug resistance (MDR) phenotype, which almost constantly expressed in HCC, is generally considered as a key cause of poor outcome in HCC chemotherapy [13, 14]. MDR is associated with overexpression of the MDR1 gene and the expression of a transmembrane glycoprotein of 150–180 kDa membrane phosphoglycoprotein (P-gp). As a drug pump, it confers cancer cell resistance to a broad range of structurally and functionally diverse chemotherapeutic drugs [15, 16]. It is well documented that MDR has frequently been associated with elevated expression level of the MDR1/P-gp in hepatocellular carcinoma cell lines [17, 18].

Protein citrullination had recently become an interest area in some types of cancer. PADI4 was a transcriptional coregulator that catalyzes the calcium-dependent conversion of specific arginine residues in proteins to citrulline, and had also been proposed to “reverse” epigenetic histone modifications [19]. PADI4 expression was also significantly detected in some non-adenocarcinoma tumors such as liver cholangiocellular carcinoma and hepatocellular carcinoma, but the levels of PADI4 expression by immunohistochemistry or western blot analysis were very different in various malignant tumor tissues and blood [20, 21]. In this study, we found that the elevated PADI4 expression is associated with chemoresistance in HCC patients with TACE after surgery. Therefore, we explore the role of PADI4 in chemoresistance of HCC cells. We found that overexpression of PADI4 in HCC cell lines could lead to the resistance to chemotherapeutic agents *in vitro* and *in vivo*.

Autophagy is an evolutionarily conserved catabolic process, which serve as a survival mechanism in a nutrient deficient environment not only for prokaryotic but also for eukaryotic cells [21, 22]. The autophagy associated pathway consists three steps: formation of autophagosome, lysosomal fusion with the autophagosome, and lysosomal degradation to produce precursor molecules, such as amino acids fatty acids and other precursor molecules, to be reutilized for maintaining cellular homeostasis and facilitating cell survival [23–26]. Autophagy has been reported to be associated with several physiological and pathological processes, including cell differentiation, tumorigenesis and adaptation to changed environmental conditions [27]. However, the role of authphagy in tumorigenesis is still controversial. On one hand, some studies reported that autophagy is essential for the survival of cancer cells not only in cancer cells but also in cancer stem cells [11, 28]. On the other hand, prolonged autophagy will lead to non-apoptotic type II programmed cell death [29, 30].

In previous studies, we have demonstrated that autophagy decreases the sensitivity of hepatoma cells to chemotherapeutic agents by affecting their apoptotic potential [11]. In addition, we have shown that autophagy activated by hypoxia mediates the tolerance of hepatocellular carcinoma cells to nutrient deprivation, which is dependent on the activity of Beclin 1 [19]. Wang, et al. found that PADI4 regulates the mTORC1 signaling pathway and PADI inhibitors are potential anticancer reagents that activate tumor suppressor gene expression alone or in combination with HDAC inhibitors [31].

Interestingly, our study indicated that HCC cells with overexpressed PADI4 were observed to undergo autophagy, which is known as a protective mechanism for cells to resist the cell toxicity from chemotherapy. Autophagy inhibitor could effectively restore the sensitivity of HCC cells to chemotherapy *in vitro* and *in vivo*. These results indicate that PADI4 could induce chemoresistance in HCC cells by leading autophagy.

## Conclusions

Taken together, PADI4 could play an important role in inducing chemoresistance in HCC cells, and the expression of PADI4 of tumor tissues in HCC patients could be used as a prognostic indicator. However, this remains a speculation and more studies are needed in the future to elucidate the exact molecular mechanisms of PADI4 in HCC.

## Electronic supplementary material

Additional file 1: Table S1: Sequence of the oligonucleotides for shRNA construct-making assays. **Table S2.** Correlations Between MDR1 Expression and Clinicopathologic Variables of HCC. (DOCX 17 KB)

Additional file 2: Figure S1: The original expression and overexpression of PADI4 in HCC cell lines: Western blot was used to analysis the expression of PADI4 in SMMC7721 and HepG2 cell lines. GAPDH expression was used as control. (TIFF 2 MB)

Additional file 3: Figure S2: Inhibition of autophagy restored the sensitivity of HCC cells to chemotherapy: SMMC-7721 and Hep-G2 cells (1×10^4^/well) that overexpressed PADI4 were cultured in a 96-well plate with an existence of 5-Fu (120μg/mL) for 24 hours. The occurrence of autophagy was inhibited by autophagy inhibitor-CQ. MTT was used to detect the viability of the cells. (TIFF 426 KB)

Additional file 4: Figure S3: The knowndown efficacy of shRNA-ATG7 and shRNA-Beclin1: Realtime PCR was employed to examine the knowndown efficacy of shRNA-ATG7 and shRNA-Beclin1 in HCC cells when cultured in hypoxia condition. (TIFF 812 KB)

## Abbreviations

PADs: Peptidylarginine deiminases
TACE: Transcatheter arterial chemoembolization
HCC: Hepatocellular carcinoma.
