# Supplementary material for: Peptidylarginine deiminase IV promotes the development of chemoresistance through inducing autophagy in hepatocellular carcinoma
Source: Cell Biosci. 2014 Aug 26;4:49. doi: 10.1186/2045-3701-4-49 (PMC4412294; doi:10.1186/2045-3701-4-49)
Supplement: Supplementary file 1 — Additional file 1: Table S1: Sequence of the oligonucleotides for shRNA construct-making assays. Table S2. Correlations Between MDR1 Expression and Clinicopathologic Variables of HCC. (DOCX 17 KB) [file 13578_2014_179_MOESM1_ESM.docx]

**Supplymentary table 1 Sequence of the oligonucleotides for shRNA construct-making assays**

| **Assays** | **Gene** | **Sequence (5’ 🡪 3’)** |
| --- | --- | --- |
| **PADI4** | Sequence 1 | CCAGAUUUUGGCUAUGUAACUTT |
|  | Sequence 2 | GACAUUGAGAGAACAUAAUUCTT |
| **ATG7** | Sequence | CCAAGGTCAAAGGACGAAGATT |
| **Beclin1** | Sequence | GCAGATGAGGAAGATCGCCTT |

**Supplymentary table 2 Correlations Between MDR1 Expression and Clinicopathologic Variables of HCC**

| **Clinicopathologic**  **Parameters** | **N** | **MDR1 expression levels** | | ***P*-Value** |
| --- | --- | --- | --- | --- |
|  |  | **Low** | **High** |  |
| **Age (y)**  ≤60  >60 | 85  35 | 26  7 | 59  28 | 0.238 |
| **Gender**  Male  Female | 90  30 | 25  8 | 65  22 | 0.906 |
| **Cirrhosis**  Presence  Absence | 81  39 | 19  14 | 62  25 | 0.153 |
| **BCLC stage**  A  B or C | 52  68 | 17  16 | 35  52 | 0.265 |
| **Tumor size (cm)**  ≤3  >3 | 62  58 | 15  18 | 47  40 | 0.402 |
| **Tumor margin**  Clear  Invovled | 45  75 | 15  18 | 30  57 | 0.268 |
| **Tumor nodule number**  Solitary  Multiple (≥2) | 48  72 | 17  16 | 31  56 | 0.113 |
| **Portal vein thrombosis**  Presence  Absence | 68  52 | 14  19 | 54  33 | 0.052 |
| **UICC TNM stage**  T1-2  T3-4 | 42  78 | 13  20 | 29  58 | 0.534 |
| **Edmondson grade**  Low(Ⅰ/Ⅱ)  High(Ⅲ/Ⅳ) | 47  73 | 16  17 | 31  56 | 0.198 |
