# Supplementary figures and images for: Peptidylarginine deiminase IV promotes the development of chemoresistance through inducing autophagy in hepatocellular carcinoma
Source: Cell Biosci. 2014 Aug 26;4:49. doi: 10.1186/2045-3701-4-49 (PMC4412294; doi:10.1186/2045-3701-4-49)

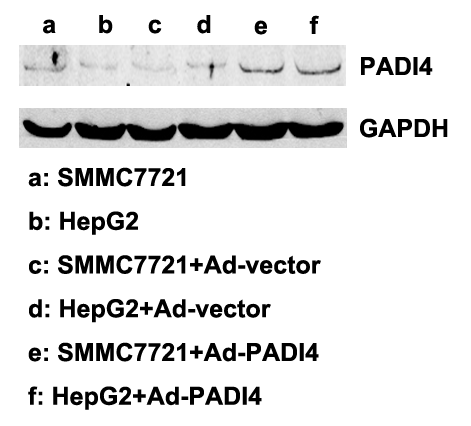

Supplement: Supplementary file 2 — Additional file 2: Figure S1: The original expression and overexpression of PADI4 in HCC cell lines: Western blot was used to analysis the expression of PADI4 in SMMC7721 and HepG2 cell lines. GAPDH expression was used as control. (TIFF 2 MB) [file 13578_2014_179_MOESM2_ESM.tiff]

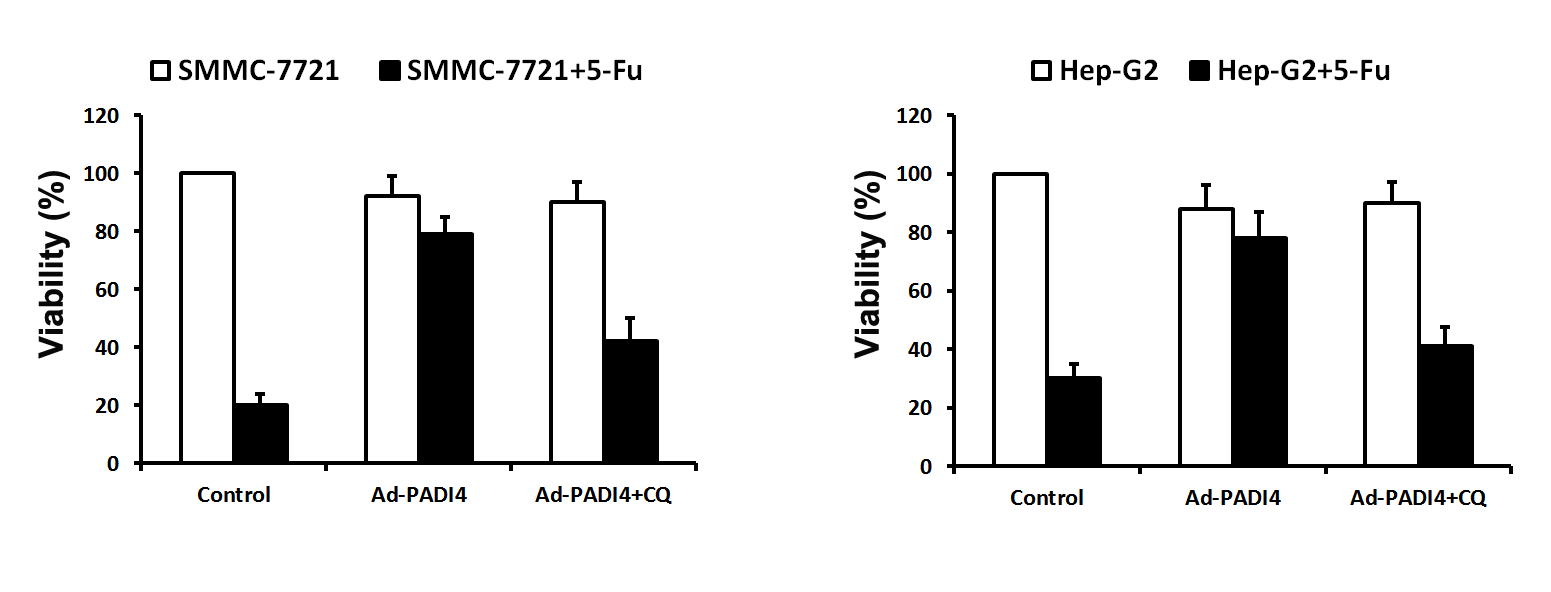

Supplement: Supplementary file 3 — Additional file 3: Figure S2: Inhibition of autophagy restored the sensitivity of HCC cells to chemotherapy: SMMC-7721 and Hep-G2 cells (1×10^4^/well) that overexpressed PADI4 were cultured in a 96-well plate with an existence of 5-Fu (120μg/mL) for 24 hours. The occurrence of autophagy was inhibited by autophagy inhibitor-CQ. MTT was used to detect the viability of the cells. (TIFF 426 KB) [file 13578_2014_179_MOESM3_ESM.tiff]

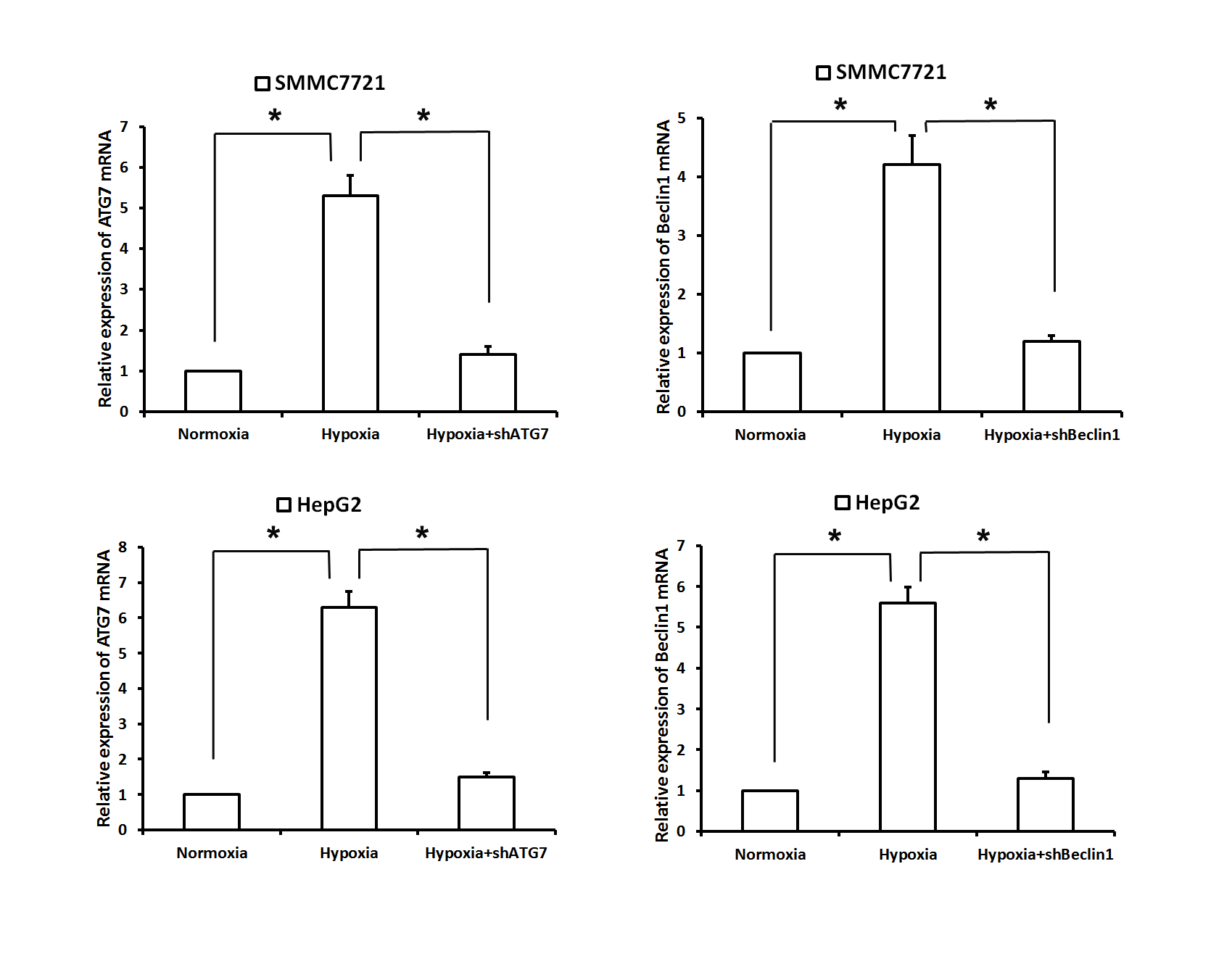

Supplement: Supplementary file 4 — Additional file 4: Figure S3: The knowndown efficacy of shRNA-ATG7 and shRNA-Beclin1: Realtime PCR was employed to examine the knowndown efficacy of shRNA-ATG7 and shRNA-Beclin1 in HCC cells when cultured in hypoxia condition. (TIFF 812 KB) [file 13578_2014_179_MOESM4_ESM.tiff]
